# Citronellal as a Promising Candidate for Alzheimer’s Disease Treatment: A Comprehensive Study on In Silico and In Vivo Anti-Acetylcholine Esterase Activity

**DOI:** 10.3390/metabo13111133

**Published:** 2023-11-04

**Authors:** Pavani K, D S. N. B. K. Prasanth, Murthy K. R. Shadakshara, Sheikh F. Ahmad, Ramanjaneyulu Seemaladinne, Mithun Rudrapal, Praveen Kumar Pasala

**Affiliations:** 1 Department of Pharmacology, Santhiram College of Pharmacy, Jawaharlal Nehru Technological University Anantapur, Nandyal 518112, Andhra Pradesh, India; pavanivemulapati25@gmail.com; 2 Department of Pharmacognosy, KVSR Siddhartha College of Pharmaceutical Sciences, Vijayawada 520010, Andhra Pradesh, India; dsnbkprasanth@gmail.com; 3 Department of Chemical Engineering, Siddaganga Institute of Technology, Tumkur 572103, Karnataka, India; krsmurthy@sit.ac.in; 4 Department of Pharmacology and Toxicology, College of Pharmacy, King Saud University, Riyadh 11451, Saudi Arabia; 5 Department of Chemistry and Biochemistry, Lamar University Beaumont, Beaumont, TX 77705, USA; rseemaladinne@gmail.com; 6 Department of Pharmaceutical Sciences, School of Biotechnology and Pharmaceutical Sciences, Vignan’s Foundation for Science, Technology & Research (Deemed to be University), Guntur 522213, Andhra Pradesh, India; drmr_pharma@vignan.ac.in; 7 Department of Pharmacology, Raghavendra Institute of Pharmaceutical Education and Research, JNTUA, Anantapuramu 515721, Andhra Pradesh, India

**Keywords:** Citronellal (CTN), acetylcholine esterase, Alzheimer’s disease (AD), molecular dynamic, antioxidant activity

## Abstract

One of the primary therapeutic approaches for managing Alzheimer’s disease (AD) involves the modulation of Acetylcholine esterase (AChE) activity to elevate acetylcholine (ACh) levels inside the brain. The current study employed computational chemistry approaches to evaluate the inhibitory effects of CTN on AChE. The docking results showed that Citronellal (CTN) and standard Donepezil (DON) have a binding affinity of −6.5 and −9.2 Kcal/mol, respectively, towards AChE. Further studies using molecular dynamics (MD) simulations were carried out on these two compounds. Binding free energy calculations and ligand-protein binding patterns suggested that CTN has a binding affinity of −12.2078. In contrast, DON has a much stronger binding relationship of −47.9969, indicating that the standard DON has a much higher binding affinity than CTN for AChE. In an in vivo study, Alzheimer-type dementia was induced in mice by scopolamine (1.5 mg/kg/day i.p) for 14 days. CTN was administered (25 and 50 mg/kg. i.p) along with scopolamine (SCO) administration. DON (0.5 mg/kg orally) was used as a reference drug. CTN administration significantly improved the mice’s behavior as evaluated by the Morris water maze test, evident from decreased escape latency to 65.4%, and in the CPS test, apparent from reduced escape latency to 69.8% compared to the positive control mice. Moreover, CTN significantly increased the activities of antioxidant enzymes such as catalase and superoxide dismutase (SOD) compared to SCO. Furthermore, CTN administration significantly decreased SCO-induced elevated AChE levels in mice. These results were supported by histopathological and in silico molecular docking studies. CTN may be a potential antioxidant and neuroprotective supplement.

## 1. Introduction

Alzheimer’s disease (AD) is a progressive and incurable neurodegenerative disorder that affects the neurons in the brain [1]. According to the World Health Organization (WHO), AD is widely recognized as a prominent contributor to the prevalence of dementia, affecting an estimated 50 million individuals globally [2].

The pathogenesis of AD is not yet completely understood, with multiple factors contributing to neuronal cell death to different extents [3,4,5,6]. Such factors may, for example, be lifestyle factors and conditions leading to upregulated production of proinflammatory cytokines [7,8], accumulation of abnormal deposits of β-amyloid peptide (Aβ) and hyperphosphorylated tau protein, in addition to widespread cell death and the loss of synapses, especially in the cholinergic system, which are considered to define the pathophysiology of AD [9].

Acetylcholinesterase plays a key role in inhibiting the transmission of nerve impulses at cholinergic synapses by catalysing the breakdown of the neurotransmitter acetylcholine. Additionally, it speeds up the clumping together of β-amyloid peptides. A significant strategy to manage Alzheimer’s disease involves maintaining the levels of acetylcholine in the synaptic cleft through inhibiting acetylcholinesterase [10,11].

Currently, four licensed medications targeting AChE are used to treat AD. These treatments include tacrine, Donepezil (DON), galantamine, and rivastigmine, as referenced in sources [12,13,14,15,16]. Regrettably, these pharmaceutical substances are recognized for their propensity to elicit several adverse effects, including disturbances in the gastrointestinal system, and they exhibit diminished bioavailability. Therefore, it is imperative to discover and cultivate AChE inhibitors, such as those derived from natural sources, to treat AD with minimal adverse effects.

Consumption of bioactive compounds derived from natural sources, including alkaloids, flavonoids, tannins, saponins, and other phytochemicals, has demonstrated promising effects. These active compounds have been identified as having significant therapeutic benefits in different applications [17,18,19,20]. Numerous pharmaceuticals produced from plants have been examined in clinical phase trials, and trials of certain compounds against diseases that pose worldwide challenges have been effectively concluded [21,22]. Most people rely solely on crude extracts to address their health concerns and need to understand the scientific basis behind their effectiveness. Numerous studies have identified and extracted bioactive compounds from natural sources. However, to fully understand the mechanisms and effects of these compounds, complete investigation is necessary to elucidate their molecular interactions.

Citronellal (CTN), a naturally occurring chemical, has garnered significant interest owing to its diverse range of pharmacological actions. This compound is a monoterpenoid aldehyde commonly present in the essential oils of various plants, including citronella, lemongrass, and cardamom [23]. CTN has been studied for its antifungal [24], antioxidant, antidepressant [25], insecticidal [26], anti-inflammatory [27], analgesic [28], anti-anxiety [29], cardioprotective [30], and antidiabetic [31] effects and in vitro investigations have shown documented AchE inhibition [32]. Despite this, there is a lack of scientific evidence supporting the effectiveness of CTN in the treatment of Alzheimer’s disease. Therefore, the current work was conducted to perform a molecular docking and MD simulation to determine the binding affinity and stability of CTN on AchE. This was followed by subsequent in vivo investigations on Alzheimer’s disease [33].

Molecular docking is highly effective for predicting ligand orientation within a protein’s location and investigating protein-ligand interactions. Molecular dynamics (MD) simulations offer comprehensive structural insights into dynamic fluctuations and conformational alterations [34]. Recently, several studies have employed molecular docking techniques to investigate the interactions and conformations of ligands with AChE targets [35]. Additionally, MD modelling has been utilized to examine the structural characteristics of AChE-ligand binding [36]. In this study, we investigated the potential medicinal properties of CTN as a therapeutic agent against AD. This investigation used an integrative strategy that combined structural modelling and experimental analysis. A comprehensive understanding of the interactions between CTN and proteins and their inhibitory effects on AChE and quantified brain AchE, antioxidant, and cognitive function in scopolamine-induced AD mice could prove advantageous in advancing drug design and development for treating AD.

## 2. Materials and Methods

### 2.1. Drug Likeliness and ADME/T Analysis

In computer-based drug development, there is growing interest in using in silico ADMET analyses [37]. ADMET analysis was used to determine the pharmacological structure in the context of drug discovery. Pharmacokinetic and drug similarity predictions for substances were conducted using the online program ADMETSAR (http://lmmd.ecust.edu.cn/admetsar1/, Accessed on 12 September 2023) [38,39]. Pharmacokinetic and drug similarity predictions were used to assess Lipinski using the DruLito program [40].

### 2.2. Molecular Docking

Molecular docking analysis was conducted using AutoDock Vina [41] software (version 2.1), focusing on the active Human AChE (PDB: 4EY7) site. The three-dimensional structures of CTN and l-dopa were obtained from PubChem [42] and subjected to energy minimization using the mmf94 force field. The X-ray crystallographic structure of the Human AChE (PDB code: 4EY7) [43] was obtained from the Protein Data Bank (PDB) server located at www.rcsb.org. The target and ligand were prepared using AutoDock Vina software, while the docking process employed the Lamarckian Genetic Algorithm (LGA) approach. The active site of the protein target was determined by creating a grid box with the dimensions of X −14.108464, Y −43.832714, and Z 27.669929 Å, with a grid spacing of 0.375 Å. The grid box was centered at 10 Å, 10 Å, and 10 Å. Following the completion of the docking process, the conformation exhibiting the lowest docking energy was selected, and the optimal pose was subsequently preserved. Ten runs were conducted using AutoDock Vina software for each ligand. Protein–ligand conformations and hydrogen bond interactions were analyzed using Discovery Studio Visualizer 2021 [44].

### 2.3. Molecular Dynamics

Molecular Dynamics (MD) simulations were conducted on a subset of protein-ligand complexes obtained from docking experiments using Gromacs-2019.4. Force-field coordinates were acquired by obtaining the ligand topology from the ATB server. The initialization of the system was performed using the steepest descent technique, followed by a 1500-step minimization phase conducted under vacuum. A Simple Point Charge (SPC) water model was utilized to dissolve the complex structures, and the facilities were positioned within a cubic periodic box of 0.5 nm. To maintain a salt concentration of 0.15 M in the intricate system, it was vital to introduce a suitable quantity of Na^+^ and Cl^-^ counter ions. The plan was prepared according to a previously published technique [45]. Following the NPT equilibration phase, a final simulation run lasting 100 ns was performed in the ensemble. During the final stage of the analysis, several GROMACS tools, specifically gmx rms, gmx rmsf, gmx gyrate, gmx sasa, and gmx bond, were used to calculate the significant structural parameters. These parameters included root-mean-square deviation (RMSD), root-mean-square fluctuations (RMSF), radius of gyration (Rg), solvent-accessible surface area (SASA), and intramolecular hydrogen bonding. This analysis was conducted for both the wild-type and mutant proteins. To obtain a comprehensive understanding of the binding free energy (ΔG binding) between the inhibitor and the protein during the simulation, the Molecular Mechanics Poisson-Boltzmann surface area (MM-PBSA) approach was employed. The binding free energy was calculated using the GROMACS software 5.1.4 tool, g_mmpbsa. The findings were obtained by examining the change in Gibbs free energy (ΔG) during the last 50 ns, which consisted of 1000 frames [46].

### 2.4. Chemicals and Software

Scopolamine hydrobromide (SCO) (Yarrow pharma, Mumbai, India), Donepezil (DON) (Alkem laboratories Ltd., Mumbai, India), CTN (SRL, Hyderabad, India), Autodock Vina, and Discovery Studio Visualizer 2021.

### 2.5. Animals and Treatment

Mice weighing 20–23 g were purchased (Vyas Labs, Hyderabad, India). The mice were housed in individual cages under controlled temperature (20 °C ± 2 °C), 12-h light/dark cycle, and specific humidified conditions (50% ± 10%). All mice were provided ad libitum access to water and a standard diet. The Santhiram College of Pharmacy approved the animal protocol used in the present study, Institutional Animal Care and Use Committee (1519/Po/Re/S/11/CPCSEA/2022/001). After one week of acclimatization, the mice (*n* = 40) were randomly divided into five groups (*n* = 8). Negative control: (Saline treatment group); Positive control: SCO (1 mg/kg, i.p, 14 days); treated groups: DON (0.5 mg/kg, p.o) + SCO (1 mg/kg, i.p, 14 days); CTN (25 mg/kg. I.P) + SCO (1 mg/kg, i.p, 14 days); CTN (50 mg/kg. I.P) + SCO (1 mg/kg, i.p, 14 days). SCO and CTN were dissolved in 0.9% sodium chloride (NaCl) and administered by *i.p*. injection to mice. The behavioral experiments were conducted 30 min after the SCO injection.

### 2.6. Morris Water Maze (MWM) Test

The MWM test is commonly employed to assess spatial learning and memory in animal models [47,48]. The water maze comprised a circular pool with dimensions of 1.0 m in diameter and 0.38 m in height. The collection was filled with black ink at a temperature of (23 ± 2 °C), reaching a depth of 25 cm. The platform remained stationary during the duration of the training period. A non-toxic dye was introduced into the water to render the platform invisible, resulting in its opaqueness. The study consisted of performing memory acquisition times, each lasting 120 s, on two occasions per day for a total of four consecutive days. There was a minimum gap of 15 min between each trial. Throughout each acquisition experiment, the animals were afforded unrestricted freedom to navigate the water and actively seek the hidden platform. After successfully identifying the platform, it was allowed to remain on it for an additional 20 s to rest. If an animal could not reach the platform within 120 s, it was gently directed toward it and then preserved on the platform for 20 s. The researchers determined the mean escape latency by measuring the time it took for each mouse to find the hidden platform. This measure was used to indicate the mice’s acquisition or learning abilities.

### 2.7. Cook’s Pole Climbing (CPC) Test

The learning and memory of the animals were evaluated by assessing the conditioned avoidance response using the Cooks pole climbing apparatus [49]. The ground part consists of grid rods that act as a shock stimulus. First, individual mice were trained, and readings were noted acquisition; the retention trail was recorded on the 0th, 7th, and 14th days. The cut-off time of 120 s was considered for evaluation [50]. Trained animals were treated with saline, CTN, or DON, and the conditioned avoidance responses were assessed.

### 2.8. Collection of Brain Samples

At the end of the behavioral experiment, animals were sacrificed by cervical decapitation under light anesthesia. Immediately after decapitation, the brain was taken out, weighed, and cleaned with isotonic saline. In 0.1 M phosphate buffer (pH 7.4), a 10% (*w*/*v*) tissue homogenate was created. The homogenates (10% *w*/*v*) were then centrifuged at 10,000× *g* for 15 min to generate the supernatant, and the resultant cloudy supernatant liquid was used to estimate brain biochemical parameters [51].

### 2.9. Brain Chemical Parameters

AChE activity (expressed as nmol/min/mg protein) was tested according to the method adopted by Ellman et al. [52]. Catalase (expressed as n moles of H_2_O_2_ consumed/minute/mg protein) was tested by the Aebi method [53]. SOD (expressed as units per mg of protein)was tested by the Nishikimi method [54]. GSH (expressed nmol/mg/Protein) was tested by the Beulter method [55]. Activities were determined according to the methods of Aebi (1984) [53], Nishikimi et al. (1972) [54], and Ohkawa et al. (1979) [56], respectively.

### 2.10. Histopathological Examination

The brains were carefully removed, rinsed with ice-cold saline, and immediately fixed with 10% neutral buffered formalin for 72 h. Samples were processed and dehydrated in serial grades of ethanol, cleared in xylene, and then infiltrated and embedded into Paraplast plus tissue embedding media. Coronal brain sections were processed for paraffin embedding, and 4 µm sections were cut by a rotatory microtome and mounted on glass slides. Cells were then stained with hematoxylin and eosin (H&E) and examined under a light microscope (Fluorescent Biological Binocular Microscope with Achromatic Objective, Los Angeles, CA, USA. Labomed, Inc.’s LB-208) [57].

### 2.11. Statistical Analysis

All results were expressed as mean ± Standard Error (SEM). Data were analyzed using one-way ANOVA and Dunnett’s *t*-test [58]. At least, at *p* < 0.05 *, *p* < 0.01 **, *p* < 0.001 *** the results were considered statistically significant.

## 3. Results

### 3.1. Drug Likeliness and ADMET Analysis

Drug likeness evaluates how much a substance’s physicochemical and structural attributes align with those often observed in established pharmaceuticals. Based on the results displayed in Table 1, CTN and DON were ideal molecules that obey Lipinski’s Rule of Five.

Computational methods for predicting ADMET properties, such as in silico forms, have become increasingly popular. These methods offer several advantages over traditional in vitro methods, including being faster, cheaper, and potentially more lifesaving [39]. In silico processes use computer models and algorithms to predict a compound’s ADMET properties based on its chemical structure and other relevant factors without animal models. This reduces the time and expense associated with traditional methods [59].

Regarding DON, the drug has been found to have toxicities such as carcinogenicity, immunotoxicity, and cytotoxicity. No toxicities were associated with CTN; its LD_50_ was reported as 505 mg/Kg, placing it in Class 4. CTN is classified as Class 5, with an LD_50_ of 2420 mg/kg. CTN was better than DON regarding solubility, and both compounds displayed better HIA and BBB. Comparing both compounds, CTN has better properties than DON (Table 2).

### 3.2. Molecular Docking

**Figure 1:**
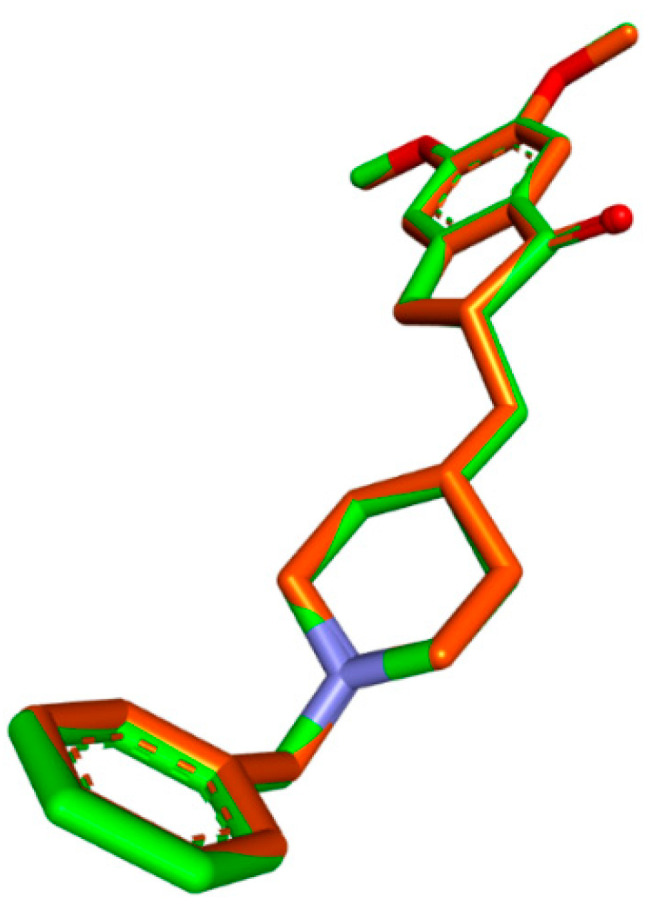
Validation of the docking algorithm by redocking the native inhibitor DON with human AChE (PDB:4EY7). Green: native crystallized pose of risperidone; Orange: Docked pose of DON.

To ensure the accuracy of our docking procedure, a re-docking study was conducted before docking any ligands to the AChE structure. One of the ligands we used, DON, has a crystal structure of 4ey7. We anchored the ligand to the AChE binding site and validated the results using RMSD values. The RMSD value between the re-docked and co-crystallized poses was 0.99658, demonstrating our docking protocol’s effectiveness and reliability (Figure 1).

**Figure 2:**
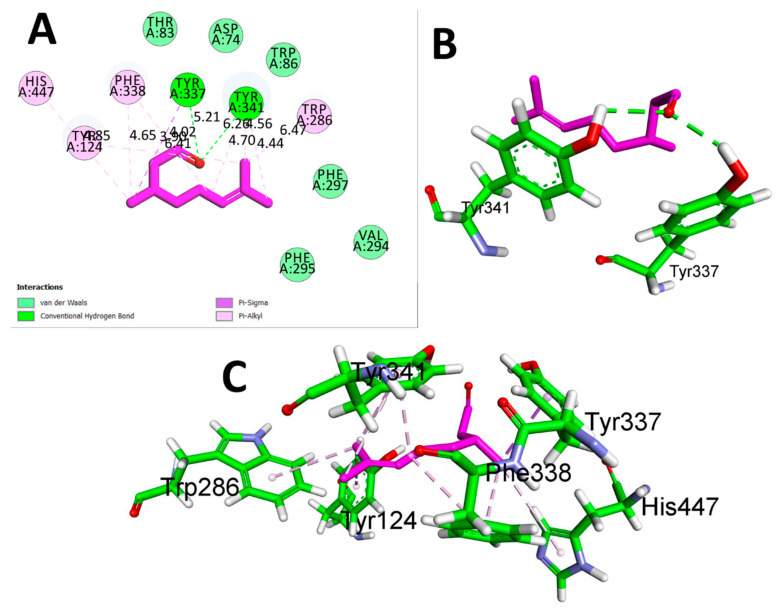
(**A**) 2D interaction view of CTN with Human AChE (PDB ID:4EY7). (**B**) 3D view of hydrogen bond interactions (**C**) 3D view of hydrophobic interactions.

CTN exhibited binding solid affinity towards the AChE enzyme, as determined by a calculated binding free energy (ΔG) of −6.5 Kcal/mol. The interaction between CTN and AChE was thoroughly analyzed, revealing the precise participation of amino acid residues and their distances in binding interactions. Hydrogen bond interactions were detected between CTN and TYR A337 (5.21 Å) and TYR A: 341 (6.26 Å). The residues TYR A:124, HIS A:447, PHE A:338, TYR A:337, TYR A:341, and TRP A:286 exhibited significant hydrophobic interactions at distances of 4.65 Å, 4.85 Å, 3.90 Å, 6.41 Å, 4.70 Å, 4.56 Å, and 4.44 Å, respectively. These findings suggest that CTN can form a stable complex with AChE by forming hydrogen bonds and hydrophobic contacts, which are believed to play a role in CTN’s anti-AChE activity (Table 3 and Figure 2).

**Figure 3:**
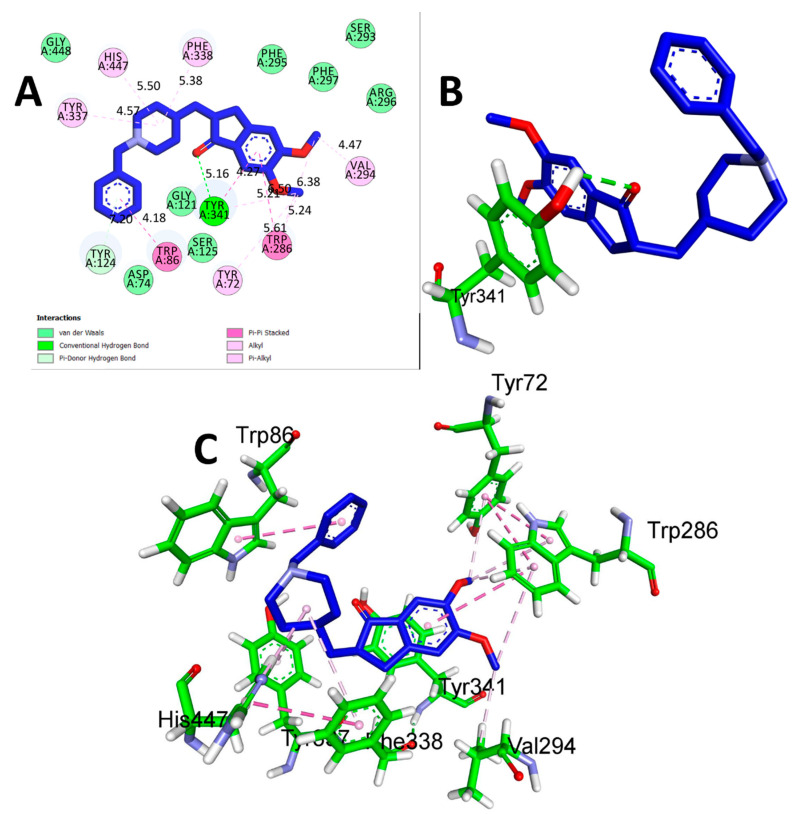
(**A**) 2D interaction of donepezil with human AChE (PDB ID:4EY7). (**B**) 3D view of hydrogen bond interactions (**C**) 3D view of hydrophobic interactions.

The results of the comparison between CTN and DON revealed that DON, the reference drug, demonstrated a significantly stronger binding affinity (−9.2 Kcal/mol) with AChE through hydrogen bonding with TYR A: 341 at a distance of 5.16 Å. Additionally, hydrophobic interactions were detected between TYR A:72 (5.61 Å), ARP A:86 (4.18 Å), TRP A:286 (5.21 Å), TYR A:337 (4.57 Å), HIS A:447 (5.50 Å), PHE A:338 (5.38 Å), and VAL A:294 (4.47 Å) (Table 3 and Figure 3). While DON displayed a greater affinity for binding, the potential benefits of CTN, a natural compound source, and its safety profile should also be considered. These factors make CTN a promising option for further research as a potential treatment for AD. The findings of this study highlight the potential of CTN as a primary compound for the development of innovative therapeutic agents for the treatment of AD.

### 3.3. Molecular Simulation Studies

We performed comprehensive all-atom molecular dynamics (MD) simulations to explore how CTN and DON complexes influence the conformational stability of human AChE protein. We performed 100-ns MD simulations for two complexes: (i) the protein complexed with CTN and (ii) the protein complexed with DON (DON). The average RMSD, RMSF, RG, and SASA values are listed in Table 4.

**Figure 4:**
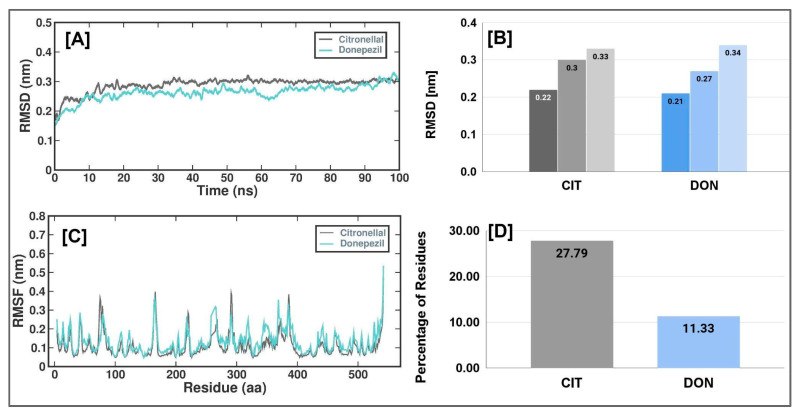
Simulation plots of CTN and DON with 4EY7 (**A**) The RMSD of the AChE and CTN/DON complex during 100 ns MD simulation, (**B**) Lowest, average, and maximum RMSD values of CTN and DON with AChE (**C**) The RMSF of the AChE and CTN/DON complex during 100 ns MD simulation, (**D**) Percentage of residues with RMSF value of <2A°.

After carefully examining the trajectories, we noted a discernible improvement in stability, commencing at the 10-nanosecond interval. Consequently, our subsequent investigation focused on the latter portion of this trajectory. The complex root-mean-square deviation (RMSD) values displayed no noteworthy differences in these observations. The RMSD values for CTN and DON were recorded as 0.22, 0.31, and 0.33 nanometers for the lowest, average, and maximum values, respectively. Similarly, the corresponding values for CTN were 0.21, 0.27, and 0.34 nanometers (Figure 4B and Table 5).

Furthermore, no substantial alterations were observed in the measured structural characteristics of CTN and DON (Figure 4A). Establishing a consistent trajectory, as evidenced by the root mean square deviation (RMSD) metric, provides a robust foundation for subsequent investigation. To assess and acquire a deeper understanding of the impact of the CTN and DON complex on flexible areas of the protein structure, we performed calculations to determine the root-mean-square fluctuation (RMSF) of a calcium residue using its position averaged across time. A cut-off value of 2 Å was employed to determine the root-mean-square change (RMSF) in the context of CTN and DON (Figure 4C). The findings of this study indicated that the CTN complex protein exhibited a flexibility of 27.79%, whereas the DON complex showed a flexibility of 11.33% (Figure 4D).

**Figure 5:**
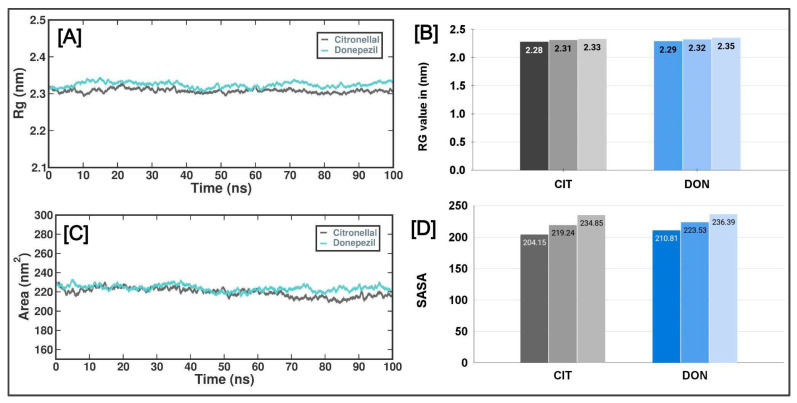
The radius of gyration (Rg) and solvent-accessible surface (SASA) regions of AChE with CTN and DON complexes. (**A**) Rg of the AChE protein and CTN/DON complex during 100 ns MD simulation. (**B**) Lowest, average, and highest Rg values of AChE_CTN and AChE_DON complexes. (**C**) The SASA values of the AChE protein and CTN/DON complex during 100 ns MD simulation, (**D**) Lowest, average, and highest SASA values of AChE_CTN and AChE_DON complexes.

The radius of gyration (Rg) indicates a molecule’s dimensions and degree of compaction. The quantity in question was determined by taking root mean square distance of the atoms in the molecule from their center of mass, with each length weighted by the mass of the respective atom. The Rg plot in Figure 5A illustrates the variations in the radius of gyration (Rg) of human AChE protein at various time intervals throughout the molecular dynamics (MD) simulation. The Rg values for the CTN and DON complexes were similar throughout the simulation. Specifically, the CTN complex had Rg scores of 2.28 nm (lowest), 2.31 nm (average), and 2.33 nm (highest). In contrast, the DON complex exhibited Rg scores of 2.29 nm (lowest), 2.32 nm (standard), and 2.35 nm (highest) (Figure 5B). Solvent-accessible surface area (SASA) refers to the quantification of the surface area of a molecule that solvent molecules can readily access. This method allows the evaluation of the degree of compactness exhibited by the hydrophobic core of a protein. Figure 5 shows the temporal variation in the solvent-accessible surface area (SASA) of the CTN and DON complexes. The SASA values of both complexes exhibited a gradual decline throughout the experiment, suggesting the progressive compaction of the hydrophobic cores of the proteins.

**Figure 6:**
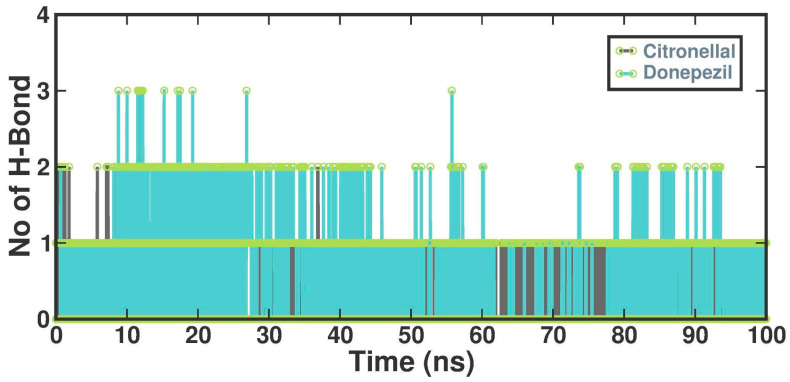
Several hydrogen bonds formed in the CTN and DON.

The number of hydrogen bonds established between CTN and DON and protein structures was also quantified using molecular dynamics (MD) simulations. This study demonstrated a diverse range of hydrogen bond formation patterns, spanning from 0 to 4 hydrogen bonds. On average, the CTN and DON complexes exhibited two hydrogen bonds (Figure 6).

#### Induction of AD

AD induced by 1.5 mg/kg/day i.p scopolamine (dissolved in normal saline) in mice for 14 days. The cerebroprotective activity and the cognitive enhancing power of CTN (25 mg/kg and 50 mg/kg/day i.p) were assessed through comprehensive behavioral, brain biochemical, and histopathological studies.

### 3.4. Behaviours Analysis

#### 3.4.1. MWM Test

**Figure 7:**
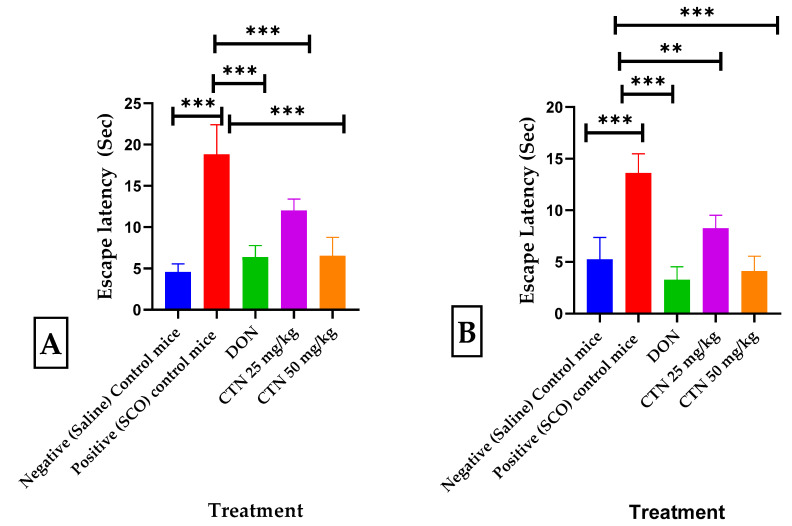
Effects of CTN on the water escape time of mice with memory impairment induced by SCO. (**A**) MWM Escape latency test (**B**) CPC Escape latency from shock. Data are expressed as means ± SEM, *n* = 8 in each group. Statistically significance *p* < 0.01 **, *p* < 0.001 ***. Comparison with Positive (SCO) control mice.

The effects of CTN on long-term and spatial memory were measured using the MWM test (F (4.35 = 59.31), *p* < 0.0001) and the swimming time for mice to find the escape platform decreased in all groups except the scopolamine-induced mice during the experimental period. SCO-treated mice exhibited a significantly longer escape latency time of 18.8 ± 1.26 (417.7%; *p* < 0.001 ***) than the negative control mice. These results demonstrated that scopolamine triggered the impairment of long-term and spatial memory. In comparison, the administration of CTN (25 mg/kg and 50 mg/kg) showed a shorter 12 ± 0.5 (36.1%; *p* < 0.001 ***), 6.5 ± 0.79 (65.4%; *p* < 0.001 ***), respectively, escape latency time compared with that of the positive control mice. In particular, CTN induced similar ELT to those of the everyday and DON 6.3 ± 0.49 (66.4%, *p* < 0.001 ***) groups Figure 7A.

#### 3.4.2. CPC Test

The escape latency time/conditional avoidance of each mouse during learning and its retention was screened by the CPC test (F (4.35 = 53.11), *p* < 0.0001). During acquisition and 0th day, no significant difference in escape latency time (ELT) was observed between positive control mice and DON treatment groups. However, on the 14th day, a considerable increase in the ELT was seen in the positive control 13.6 ± 0.65 (261.1%, *p* < 0.001 ***) compared to the negative control mice 5.25 ± 0.75 (*p* < 0.001 ***), while treatment with DON, CTN (25 mg/kg), or CTN (50 mg/kg) decreased the ELT 3.27 ± 0.45 (75.9% *p* < 0.001 ***), 8.25 ± 0.45 (39.8%; *p* < 0.01 ***) and 4.1 ± 0.52 (69.8%; *p* < 0.001 ***), respectively, when compared to the positive control mice (Figure 7B).

### 3.5. Brain Biochemical Analysis

**Figure 8:**
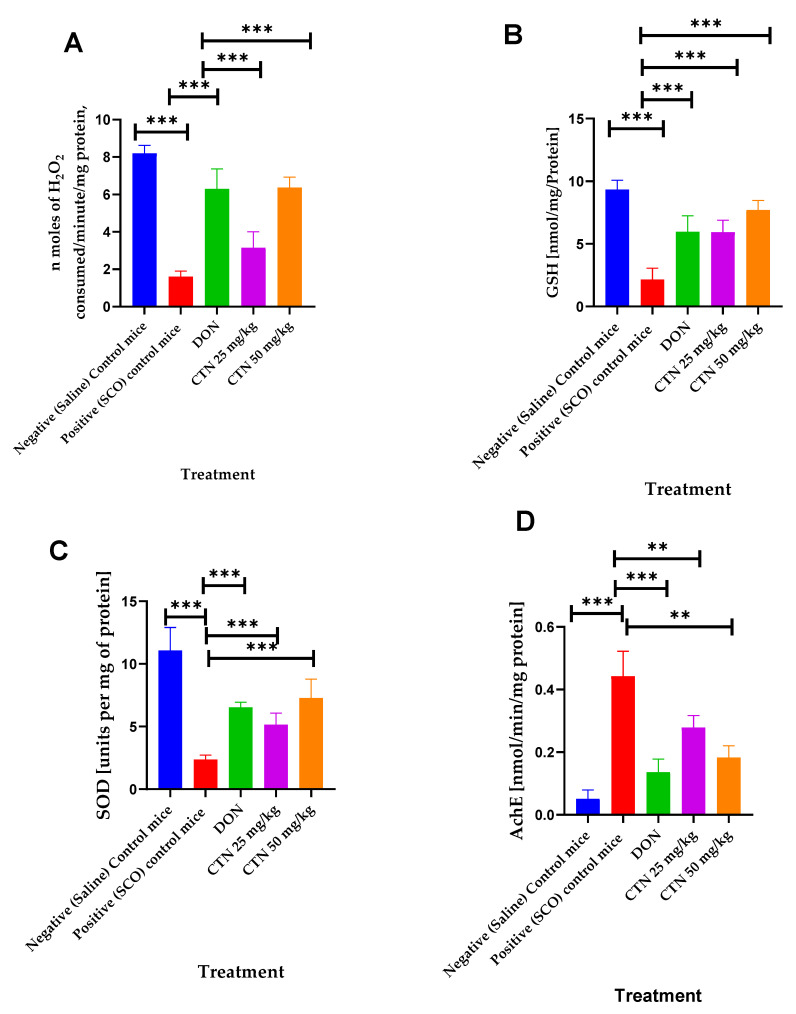
Effects of CTN on brain chemical parameters in cognitively impaired mice induced by SCO. (**A**) Catalase activity (**B**) GSH activity (**C**) SOD activity (**D**) AChE activity. Data are expressed as means ± SEM, n = 7 in each group. Statistically significance *p* < 0.01 **, *p* < 0.001 ***. Comparison with Positive (SCO) control mice.

Effect of CTN on brain catalase revealed by administration of SCO exhibited a significantly decreased brain catalase activity (1.6 ± 0.104, *p* < 0.001 ***) when compared with the negative control mice (8.19 ± 0.15). Nevertheless, the administration of CTN 25 mg/kg, CTN 50 mg/kg, and DON significantly increased the catalase activity to 3.15 ± 0.3 (*p* < 0.001 ***), 6.36 ± 0.19 (*p* < 0.001 ***), 6.29 ± 0.37 (*p* < 0.001 ***), respectively, compared with the positive control mice (F (4, 30) = 102.7, *p* < 0.0001) (Figure 8A).

The effect of CTN on brain GSH revealed by administration of SCO exhibited a significantly decreased brain GSH activity (0.34 ± 0.08, *p* < 0.001 ***) compared with the negative control mice (3.2 ± 0.09). Nevertheless, the administration of CTN 25 mg/kg, CTN 50 mg/kg, and DON significantly increased the GSH activity to 1.65 ± 0.26 (*p* < 0.001 ***), 2.02 ± 0.29 (*p* < 0.001 ***), 2.36 ± 0.30 (*p* < 0.001 ***), respectively, compared with the positive control mice (F (4, 30) = 17.91, *p* < 0.0001) (Figure 8B).

The effect of CTN on brain SOD revealed by administration of SCO exhibited a significantly decreased brain SOD activity (2.36 ± 012, *p* < 0.001 ***) compared with the negative control mice (11.07 ± 0.64). Nevertheless, the administration of CTN 25 mg/kg, CTN 50 mg/kg, and DON significantly increased the SOD activity to 5.15 ± 0.32 (*p* < 0.001 ***), 7.28 ± 0.53 (*p* < 0.001 ***), 6.53 ± 0.14 (*p* < 0.001 ***), respectively, compared with the positive control mice (F (4, 30) = 51.95, *p* < 0.0001) (Figure 8C).

The effect of CTN on brain AChE revealed by administration of SCO exhibited a significant rise in brain AChE activity (0.44 ± 0.02, *p* < 0.001 ***) when compared with the negative control mice (0.051 ±0.0098). Nevertheless, the administration of CTN 25 mg/kg, CTN 50 mg/kg, and DON significantly suppressed the AChE activity to 0.27 ± 0.031 (*p* < 0.01 **), 0.18 ± 0.01 (*p* < 0.01 **), 0.13 ± 0.01 (*p* < 0.001 ***), respectively, compared with the positive control mice (F (5, 30) = 22.51, *p* < 0.0001) (Figure 8D) (Table 6).

### 3.6. Histopathology Studies

**Figure 9:**
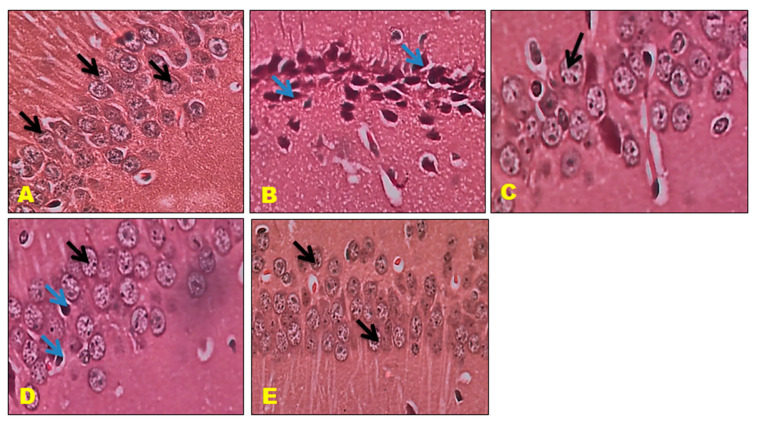
Effect of CTN on CA 1 region of AD mice (**A**) Negative Control mice exhibit closely packed cell bodies of the pyramidal neurons (black arrow) that are regularly arranged in 3 to 4 rows and appear small with vesicular nuclei, prominent nucleoli, and scanty cytoplasm. (**B**) Positive (SCO) control Mice CA1 region exhibits most of the cell bodies of the pyramidal neurons are disarranged and loosely packed; they appear dark, skinny, and have pyknotic nuclei (Blue arrow). (**C**) DON treatment CA 1 region exhibits pyramidal cell bodies that appear somewhat regularly arranged and loosely packed; few of them are shrunken with pyknotic nuclei (black arrow), (**D**) CTN 25 mg/kg treated mice DG region exhibited well-defined granule cell bodies (black arrow), still appeared pyknotic nuclei in GCL (Blue arrow) (**E**) CTN (50 mg/kg) treated mice CA1 showed packed, prominent cell body with nuclei arranged in 3 to 4 rows when compared to CTN (25 mg/kg) treated mice (Black arrow).

**Figure 10:**
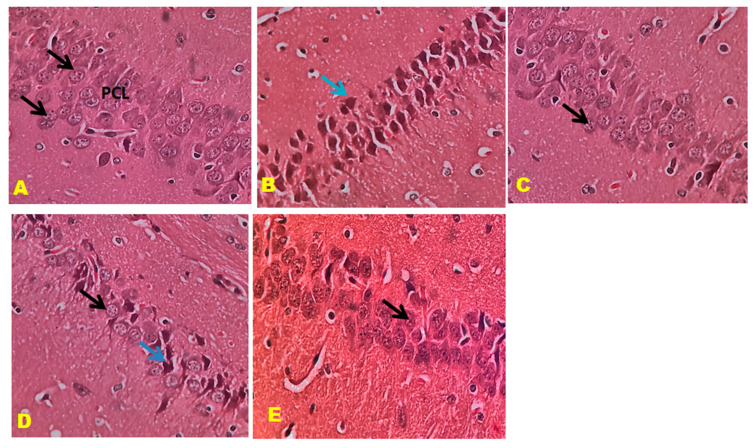
Effect of CTN on CA 3 region of AD mice (**A**) Negative control mice showed pyramidal cell bodies with large rounded vesicular nuclei and prominent nucleoli (Black arrow). (**B**) Positive (SCO) control mice showed pyramidal cell bodies disorganized and appeared darkly shrunken with pyknotic nuclei (blue arrow). (**C**) DON-treated pyramidal cell bodies containing dense large cell bodies with prominent nuclei (Black arrow). (**D**) CTN (25 mg/kg) treatment CA3 region reveals disorganized and loosely packed pyramidal cell bodies (Black arrow), some of them appear darkly shrunken with pyknotic nuclei (Blue arrow). (**E**) CTN (25 mg/kg) treatment CA3 region reveals packed pyramidal cell bodies, absence of shrunken cells results similar to DON treatment (Black arrow).

**Figure 11:**
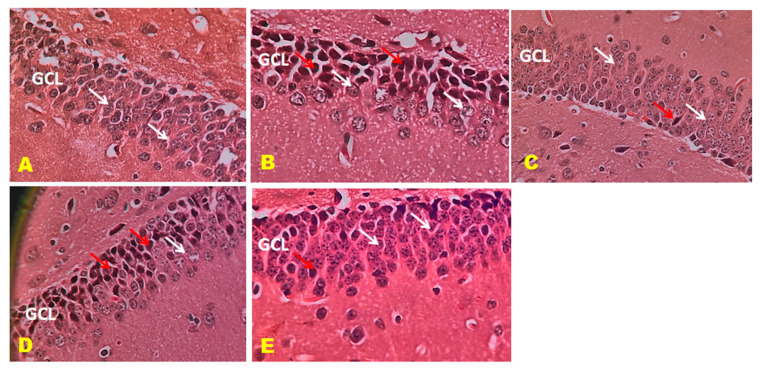
Effect of CTN on dentate gyrus (DG) region of AD mice. DG reveals well-defined three layers: ML, granule cell layer (GCL), and POL. (**A**) Positive control mice GCL reveals rounded to oval granule cell body aggregation (White arrow). (**B**) Positive (SCO) control mice GCL displays dark, shrunken granule cell bodies having pyknotic nuclei (Red arrow). Few standard granule cell bodies (White arrow) in GCL. (**C**) DON-treated DG display legal granule cell bodies (White arrow), and few appear shrunken (Red arrow). Few are dark with pyknotic nuclei in GCL. (**D**) CTN 25 mg/kg treated mice DG region exhibited well-defined granule cell bodies (White arrow), still appeared pyknotic nuclei in GCL (Red arrow) (**E**) CTN 50 mg/kg treated mice DG region exhibited well-defined granule cell bodies (White arrow), few pyknotic nuclei similar to normal and DON treated mice compared to CTN (25 mg/kg) mice (Red arrow).

The control mice revealed no histopathological alterations, with typical histological features of the hippocampus CA1, CA3, and DG regions showing intact pyramidal neurons in the brain hippocampus region. The positive control mice group histogram indicated severe damage confirmed by the appearance of dark, skinny, and pyknotic nuclei. However, CTN 50 mg/kg and standard DON-treated mice hippocampus CA1, CA3, and DG regions were significantly damaged compared to CTN 25 mg/kg treated mice on SCO-treated mice. The histopathology of the brain hippocampus at 40× magnification is shown in Figure 9, Figure 10 and Figure 11.

## 4. Discussion

Neurodegenerative diseases pose significant health challenges that impose substantial cost burdens on ageing and elderly populations [39]. The existing literature mainly indicates that individuals with AD commonly have impairment in cholinergic activity. This impairment of the cholinergic system leads to a reduction in acetylcholine (ACh) levels, primarily caused by the activation of acetylcholine esterase (AchE). The dysregulation of the cholinergic system is considered a significant contributor to the development of dementia in AD. Therefore, the current research on AD is mainly centered around the therapeutic approach of inhibiting AChE to address memory and cognitive impairment [60,61]. Therefore, the present study aims to investigate CTN’s neuroprotective and cognitive effects by targeting AChE in both in silico and in vivo.

The term “drug-likeness” refers to a set of molecular properties and structural features that determine whether a particular compound has the potential to be a drug. While in-vivo evaluations of a compound’s drug-like properties are costly and time-consuming, in-silico approaches are more cost-effective and rapid. Our studies revealed that all the compounds analyzed adhered to Lipinski’s RO5 criteria. Additionally, the ADMET profiles of these CTNs showed lower values, which indicates better drug likeliness. The partition coefficient (logPo/w) deals of these compounds were found to be correlated with the absorption potential and distribution rate of drugs within the body. The ADMET analysis revealed that all the compounds fall within the acceptable range for drug likeliness, making them potential drug-like molecules that could inhibit the proteins involved in AD. Both the combination CTN and DON fulfilled the Lipinskis RO5.

The pharmacokinetics and toxicity of phytoconstituents with pharmacological importance were predicted using the Protox II server, with toxicity evaluated in terms of LD50 values ranging from less than 50 mg/kg for Class I compounds, between 50 to 500 mg/kg for Class II compounds, between 500 to 5000 mg/kg for Class III compounds, and greater than 5000 mg/kg for Class IV compounds. Classes I, II, and III have lower toxicity, while Class IV shows no toxicity. In our study, CTN was classified in Class 5 with an LD_50_ value of 2420 mg/Kg; in the case of DON, it is placed in Class 4 with an LD_50_ of 505. DON displayed toxicities related to carcinogenicity, immunotoxicity, and cytotoxicity, but these were not observed in the case of CTN. Previous studies on DON have documented numerous adverse effects, including altered mental status, bradycardia, and cardiovascular autonomic control issues [62,63]. Based on this study, CTN was safer than DON regarding ADMET analysis as it is of natural origin and considered safe.

Molecular docking is a valuable tool employed in computational analysis for drug development, used to determine the binding energy between a ligand and a receptor [64]. The molecular docking study revealed that DON, the standard drug, had a better binding energy of −9.2 Kcal/mol than CTN (−6.5 Kcal/mol). This study also showed that the amino acids Tyr337, Tyr341, and His447 are shared between DON and CTN. Previous studies have reported that these amino acids play a vital role in the inhibition of Ache [65,66,67]. This suggests that CTN has excellent potential for inhibiting AChE and treating Alzheimer’s.

Molecular Dynamics (DM) is a computational method used to simulate the dynamic behavior of molecular systems over time, treating all the entities in the simulation box as flexible. This method was used to infer the motions and flexibility of the protein, which affects the interaction dynamics of the complexes. The analysis of RMSD is crucial in MD trajectory and shows the difference between the backbone of a given receptor from its initial structural conformation to its final state. The MD trajectory analysis showed the system’s stability and the drug molecules’ interaction in the simulation event. The CTN and DON had a stable conformation throughout the 100 ns simulations via RMSD, RMSF, RG, and SASA analysis [68,69].

The MM-GBSA was thoroughly examined to determine all the binding free energies, which confirmed the affirmation of the outcome regarding solvation, VDW components, and hydrophobic interactions regarding DG binding. The Prime MMGBSA was computed in conjunction with CIT-AChE and DON. The obtained free energies of the CTN and DON had a binding affinity of −12.2078 and −47.9969 kcal/mol, respectively.

Scopolamine-induced dementia has been widely employed in assessing putative therapeutic interventions for treating AD [39,48]. The study conducted by MT et al. [70] demonstrated that the administration of scopolamine in mice brains impacts the expression of many genes related to muscarinic receptor signaling pathways, apoptosis, and cell differentiation. Therefore, the degradation and malfunction of cortical cholinergic neurons are intimately linked to AD-related cognitive abnormalities, resulting in significant memory impairment in animals and humans [49]. In the present work, intraperitoneal (i.p.) administration of scopolamine resulted in a notable elevation in escape latency time (ELT) in both the MWM and the CPC tests. This was accompanied by an increased brain AChE activity and prominent histopathological changes in the brain. The administration of scopolamine resulted in the modification of behavior, neurochemical composition, and histological characteristics. However, these effects were mitigated when mice were pre-treated with DON and CTN. Previous studies have found comparable findings concerning the impacts of scopolamine [50,51] and DON [50,52].

There is a strong correlation between the extent of dementia and the severity of the neuropathologic characteristics of AD with the loss of cholinergic innervation [49]. Cholinergic transmission is terminated chiefly through the action of AChE, an enzyme responsible for the breakdown of acetylcholine (ACh), a crucial neurotransmitter, within the cholinergic neurons [53]. It has been observed that scopolamine can stimulate an elevation in AChE activity [54]. Previous studies have documented the potential of AChE inhibitors to enhance cognitive performance in mice administered scopolamine [54,55]. The present investigation demonstrated that the administration of CTN effectively suppressed the elevation of AChE activity induced by scopolamine in the brain samples obtained following the completion of the behavioral tests. According to the literature, evidence suggests that reduced AChE activity in the brain is linked to enhanced cognitive functioning [56,57]. Therefore, the findings of this study indicate that the administration of CTN has a positive impact on cognitive function, namely in terms of learning and memory. This effect is attributed, at least in part, to the suppression of AChE activity within the brain.

The occurrence of oxidative stress triggers lipid peroxidation, accompanied by a reduction in glutathione (GSH) levels and antioxidant enzyme activity. This cascade of events ultimately damages cholinergic neurons and subsequent cognitive impairments [58]. The administration of SCO has been found to induce oxidative stress, which is considered one of the primary mechanisms responsible for cell damage [39]. In the present investigation, the administration of SCO resulted in the induction of oxidative stress, as evidenced by a notable reduction in the levels of crucial brain antioxidant defenses, namely glutathione (GSH), superoxide dismutase (SOD), and catalase (CAT) activities. The administration of DON significantly elevates brain GSH, SOD, and CAT activity, suggesting augmentation of antioxidant mechanisms within the brain. The outcomes in question were pertinent to prior research [71]. The present investigation showed that administering CTN at a dosage of 50 mg/kg resulted in a considerable augmentation of antioxidant activity. This enhancement was evident across all evaluated parameters, including brain GSH, SOD, and CAT activity. The histological analysis of sections obtained from the SCO group demonstrated the presence of neurodegeneration, characterized by a significant reduction in the number of neurons. Conversely, treatment with DON exhibited limited neurodegeneration and neuronal loss. The groups treated with CTN (50 mg/kg) showed significant neuroprotection, as indicated by intact neurons and negligible neurodegeneration.

## 5. Conclusions

CTN was examined for its ability to inhibit AChE through molecular docking to treat Alzheimer’s disease. CTN and DON demonstrated better binding affinity in molecular docking studies. To assess the stability of docked CTN-AChE and DON-AChE complexes, MD simulations were conducted using Gromacs-2019.4. MM-PBSA binding free energies (ΔGbinding) indicate that CTN and DON have a binding affinity of −12.207 and −47.9969 Kcal/mol, respectively, for the AChE target. In vivo investigations have demonstrated that CTN therapy positively impacts cognitive function in mice with cognitive impairment induced by SCO. This improvement is attributed to AChE inhibition and the regulation of antioxidant pathways. The research employed the MWM and CPC techniques to illustrate the effects of the SCO on memory processes. The study findings exhibit comparability with the clinically sanctioned compound DON. Further research can be conducted to determine the impact of CTN on the phosphorylation of tau proteins and the aggregation of β-amyloid peptides along with brain bioavailability of CTN.

## Figures and Tables

**Table 1:** Drug Likeliness of the CTN.

Compound	MW	logp	Alogp	HBA	HBD	TPSA	AMR
CTN	154.14	3.591	2.043	1	0	17.07	48.38
DON	349.99	2.633	0.364	4	0	38.77	115.79

**Table 2:** ADME/T analysis of CTN.

Phytocompound	Parameters		Values
CTN	Swiss ADME	log P o/w	3.83
		Water Solubility	Soluble
		GI Absorption	High
		Lipinski Rule	Yes
		Veber’s Rule	Yes
		PAINS Alert	0
		TPSA	17.07
	ADMETSAR	HIA	0.9905
		CaCO2	0.7608
		BBB	0.9664
		CYP1A2	0.6175
		CYP2C19	0.9168
		CYP2C9	0.9225
		CYP2D6	0.9572
	PROTOX-II	LD_50_ (mg/kg)	2420 (Class 5)
		Hepatotoxicity	Inactive
		Carcinogenicity	Inactive
		Immunotoxicity	Inactive
		Mutagenicity	Inactive
		Cytotoxicity	Inactive
DON	Swiss ADME	log P o/w	3.92
		Water Solubility	Moderately Soluble
		GI Absorption	High
		Lipinski Rule	Yes
		Veber’s Rule	Yes
		PAINS Alert	0
		TPSA	38.77
	ADMETSAR	HIA	0.9966
		CaCO_2_	0.7742
		BBB	0.9953
		CYP1A2	0.5072
		CYP2C19	0.8356
		CYP2C9	0.8189
		CYP2D6	0.8919
	PROTOX-II	LD_50_ (mg/kg)	505 (Class 4)
		Hepatotoxicity	Inactive
		Carcinogenicity	Active
		Immunotoxicity	Active
		Mutagenicity	Inactive
		Cytotoxicity	Active

**Table 3:** Binding energies and interaction details of CTN and L-Dopa with AChE (PDB: 4EY7).

Ligands	Binding Affinity, ΔG (Kcal/mol)	Amino Acids Involved and Distance (Å)	
		Hydrogen-Bond Interactions	Hydrophobic Interactions
CTN	−6.5	TYR A:337 (5.21), TYR A:341 (6.26)	TYR A:124 (4.65), HIS A:447 (4.85), PHE A:338 (3.90), TYR A:337 (6.41), TYR A:341 (4.70,4.56), TRP A:286 (4.44)
DON	−9.2	TYR A:341 (5.16)	TYR A:72 (5.61), ARP A:86 (4.18), TRP A:286 (5.21), TYR A:337 (4.57), HIS A:447 (5.50), PHE A:338 (5.38), VAL A:294 (4.47)

**Table 4:** Calculated parameters (average values) for all systems based on the 100-ns MD simulation.

S. No.	Protein	RMSD (nm)	RMSF (nm)	Rg (nm)	SASA (nm 2)
1	CTN	0.3	1.11	2.31	219.2
2	DON	0.27	1.31	2.32	223.1

**Table 5:** Active binding pocket MMPBSA.

Binding Site Residues (aa)	kJ/mol	Binding Site Residues (aa)	kJ/mol
TYR-77	0.9165	TYR-72	−2.2201
PRO-78	0.1434	VAL-73	−3.3021
GLY-79	−1.2396	ASP-74	−3.4861
THR-83	0.5039	THR-75	0.1518
GLU-84	−3.5967	LEU-76	−5.9723
TRP-86	−2.1014	TRP-86	−2.3365
SER-125	1.1953	THR-83	0.0273
TRP-286	−3.5095	ASN-87	4.2641
LEU-289	−0.361	GLY-121	−1.8082
GLN-291	−0.005	GLY-122	0.0616
GLU-292	−0.9968	TYR-124	−6.2327
PHE-295	1.1175	SER-125	1.5916
ARG-296	6.215	TRP-286	−5.4894
PHE-297	−1.9667	LEU-289	−3.0761
PHE-338	−2.0933	PRO-290	−0.3583
TYR-341	−1.3473	GLN-291	−0.821
GLY-122	0.3881	GLU-292	−5.3739
PHE-123	−1.3959	SER-293	−1.0867
TYR-124	−4.0743	PHE-295	0.6986
	−12.2078	ARG-296	1.5049
		PHE-297	−4.7205
		PHE-338	−4.8894
		TYR-341	0.8496
		GLY-342	−2.329
		ALA-343	−2.5718
		PRO-344	−1.0723
			−47.9969

**Table 6:** Effects of CTN on brain chemical parameters in cognitively impaired mice induced by SCO.

Treatment	CAT (n Moles of H_2_O_2_ Consumed/Minute/mg Protein)	GSH (nmol/mg/Protein)	SOD (Units per mg of Protein)	AchE (nmol/min/mg Protein)
Negative control	8.19 ±0.15	3.2 ± 0.09	11.07 ± 0.64	0.051 ± 0.0098
Positive control	1.6 ± 0.104 ***	0.34 ± 0.08 ***	2.36 ± 012 ***	0.44 ± 0.02 ***
DON (0.5 mg/kg, p.o) + SCO (1 mg/kg, i.p, 14 days)	6.29 ± 0.37 ***	2.36 ± 0.30 ***	6.53 ± 0.14 ***	0.13 ± 0.01 ***
CTN (25 mg/kg. I.P) + SCO (1 mg/kg, i.p, 14 days)	3.15 ± 0.3 ***	1.65 ± 0.26 ***	5.15 ± 0.32 ***	0.27 ± 0.031 **
CTN (50 mg/kg. I.P) + SCO (1 mg/kg, i.p, 14 days)	6.36 ± 0.19 ***	2.02 ± 0.29 ***	7.28 ± 0.53 ***	0.18 ± 0.01 **

All the results were expressed as mean ± Standard Error (SEM). Data were analyzed using one-way ANOVA and Dunnett’s *t*-test. Statistically significance *p* < 0.01 **, *p* < 0.001 ***. Comparison with Positive (SCO) control mice.

## Data Availability

No new data were created or analyzed in this study. Data sharing is not applicable to this article.
